# Toward a new tool for risk-assessment: recidivism of child sexual abuse materials in men under voluntary treatment

**DOI:** 10.3389/fpsyg.2026.1764052

**Published:** 2026-06-30

**Authors:** Jan S. Pellowski, Ralf Bergner-Köther, Stefanie Schmidt, Lasse Peschka, Michael Hock, Peer Briken, Fritjof von Franqué

**Affiliations:** 1 Institute for Sex Research, Sexual Medicine and Forensic Psychiatry, University Medical Center Hamburg-Eppendorf, Hamburg, Germany; 2 Department for Sexual Medicine, Psychiatric Outpatient Clinic, Sozialstiftung Bamberg, Bamberg, Germany; 3 Department of Psychology, University of Bamberg, Bamberg, Germany

**Keywords:** CASIC, child pornography, child sexual exploitation material, CPORT, dark field, do not offend, recidivism, risk assessment

## Abstract

**Introduction:**

Currently, risk assessment instruments are only valid for people who have already been convicted of a criminal offense. These tools are not applicable in the case of people who present themselves in a therapeutic context and with concerns about reusing child sexual abuse material (CSAM), but without court order. This raises the question of how professionals can appropriately assess the risk of recidivism for this group of people. Following an exploratory approach, this study aims to identify key variables for the future development of a risk assessment instrument specifically designed for CSAM users in nonmandated, prevention-oriented settings.

**Methods:**

The study examined 131 male persons from two sites of the Do Not Offend (DNO) network (German translation: *Kein Täter werden*), who had used CSAM in their history. The instruments STATIC-C and CPORT/CASIC were applied exclusively based on the Information provided by the participants at the time of admission and in part retrospectively. The evaluation period was examined with regard to the self-reported CSAM recidivism.

**Results:**

Only the CASIC was able to predict the criterion, while there was no significant relationship with the STATIC-C and the CPORT without the CASIC. Using the external criterion-related strategy of scale construction, the items *No children* (not having children of one’s own) and *Previous undetected use of CSAM* as well as the CASIC items *Never married*, *Interest in child pornography spanned two or more years* and *Possessed child pornography videos* and the CPORT item *Admission or diagnosis of sexual interest in children (clinical diagnosis)* were identified as promising for future scale construction.

**Discussion:**

The results are discussed with regard to their clinical significance and the validity of a new instrument.

## 1 Introduction

Child sexual abuse material (e.g., images and videos) can be defined as photorealistic material (including AI-generated media) depicting sexual activities with at least one child (under the age of 14, as stated in the German law) or depicting children fully or partially unclothed or showing their genitals in a sexually suggestive manner. This definition largely overlaps with internationally used definitions (International Centre for Missing and Exploited Children, 2023; Seto, 2025). Law in most jurisdictions prosecutes the distribution, acquisition, and possession of these materials (International Centre for Missing and Exploited Children, 2023). Such materials are often referred to as child pornography. However, this term has been criticized (Seto, 2025) since sexual activities of adults with children cannot be consensual, which could be suggested by the term pornography (in analogy to adult pornography). For this reason, we will use the term child sexual abuse material (CSAM) in the following.^1^

Evidence suggests that CSAM is more frequently used and more accessible than ever before: Data from INTERPOL (International Child Sexual Exploitation Database, n.d.) highlights the global scale, with over 4.9 million instances identified involving approximately 18,300 distinct user accounts. This surge is mirrored in recent reporting trends: in 2024, electronic service providers submitted over 20 million reports to the CyberTipline, involving nearly 63 million files—establishing CSAM as the primary category of reported online exploitation (CyberTipeline Reports, 2024). This trajectory is also reflected in judicial trends; for instance, U.S. federal prosecutions for CSAM-related offenses nearly doubled within a decade (Adams and Flynn, 2017). Furthermore, anonymous surveys indicate a significant dark field, with 1.7% of men admitting to having viewed CSAM (Dombert et al., 2016). These figures increase significantly to 71%–79%, when participants were recruited in specific prevention contexts (Kuhle et al., 2015; von Franqué et al., 2023).

The extent to which people who have used CSAM, use it *again* is unclear. Seto et al. (2011) found a recidivism rate of 3.4% for people convicted of using CSAM, with a follow-up period ranging from one and a half to 6 years, most follow-up periods being less than 4 years. A recently published meta-analysis found a similar CSAM recidivism rate of 4.1% (95% CI = 3.8, 4.4) after an average of 5-year follow-up and based on 21 studies with a total of 13,522 subjects (Baskurt et al., 2025). However, both studies may not reflect the technical possibilities (e.g., using a Tor browser) for evading police detection. Interestingly, von Franqué et al. (2023) found a significantly higher recidivism rate of 39% with a time at risk phase of around 28 months in a study of participants in a voluntary prevention context. Most of the people in the sample had not yet been convicted of a criminal offense. These results could indicate that a large proportion of CSAM re-use is not officially registered. To address this significant gap in prevention and intercept such undetected usage, the German prevention network *Do Not Offend* (DNO; German: *Kein Täter werden*) was established. Operating within Germany’s unique legal framework (Briken et al., 2019), the network provides integrative treatment for individuals who, *inter alia*, have a history of CSAM use but wish to discontinue. While some participants may have prior convictions for sexual offenses, there were no active court orders or pending police investigations at the time of their inclusion. Consequently, their participation was non-mandated and occurred outside of the criminal justice system. Throughout this manuscript, we refer to this group as individuals in voluntary treatment or as a non-forensic sample (for further information on the network and the associated prevention program, see the Background section).

To support this treatment objective, it would be helpful to be able to identify individuals who are at particularly high risk of CSAM recidivism within such a voluntary prevention framework. This question should be relevant for prevention efforts if service providers want to structure their treatment in line with the established Risk-Need-Responsivity model (RNR model) for the successful rehabilitation of convicted persons (Bonta and Andrews, 2023) — even though the RNR model has not yet been validated for people in a voluntary prevention context. According to the risk principle, rehabilitative resources should be allocated proportionally to an individual’s level of risk: higher-risk clients require more intensive intervention. This principle rests on the foundational assumption that the risk of recidivism can be accurately measured (Bonta and Andrews, 2023) using a variety of specialized risk assessment instruments tailored to specific offense types (Douglas and Otto, 2020). The validity of the risk principle has been supported for the forensic context by various studies (e.g., Wormith and Zidenberg, 2018). After considerable efforts to adapt the RNR model for individuals who have committed sexual offenses, research has identified specific risk factors with demonstrated predictive validity (Seto et al., 2023), and meta-analytic evidence by Hanson et al. (2009) showed that treatment programs adhering to RNR principles significantly reduced recidivism rates (10.9% in treatment groups vs. 19.2% in control groups). Consequently, Hanson et al. (2009) concluded that RNR principles should be central to the design and implementation of interventions for this population. While the RNR model is regarded as the most extensively researched and scientifically supported framework to date, its status has recently become the subject of intense academic debate. Fazel et al. (2024) challenged the framework, referring to poor methodological quality across studies and potential allegiance bias among its developers. However, in a recent commentary, McGuire et al. (2025) rejected these conclusions as scientifically unjustified and misleading, arguing that the review relied on an inappropriately narrow selection of studies and unsuitable appraisal methods. Although both sides acknowledge certain research gaps, McGuire et al. (2025) maintain that the RNR model remains a robust and essential framework, warning that its dismissal would be unwarranted.

In accordance with the RNR model (Bonta and Andrews, 2023) and notwithstanding the aforementioned controversy, we contend that the effective allocation of rehabilitative resources depends on accurate risk differentiation. Consequently, the use of validated risk assessment instruments is not merely optional but a fundamental prerequisite for implementing the risk principle. Building on this rationale, the present study examines how such assessments can be designed for individuals within a voluntary prevention context to facilitate the implementation of the risk principle in a non-forensic population.

The Child Pornography Offender Risk Tool (CPORT; Seto and Eke, 2015) is an instrument for measuring the static risk of any sexual recidivism (i.e., sexual contact offenses as well as CSAM and exhibitionist offenses) among adult males with a conviction for possession of so-called child pornography (Seto and Eke, 2015). Users rate subjects on seven items. The instrument demonstrated a significant relationship with sexual recidivism in its original development sample (AUC = 0.74, 95% CI = 0.63, 0.84, *N* = 266; Seto and Eke, 2015). However, the generalizability of these findings to specific subpopulations has been a subject of ongoing discussion. Initial evaluations indicated that the CPORT did not significantly predict CSAM re-use within the subsample of individuals with a history of exclusive CSAM use (i.e., CSAM use with no other forms of sexual delinquent behavior; AUC = 0.63, 95% CI = 0.41, 0.86). This might have led Scurich and Krauss (2023) argue that the empirical evidence for the CPORT’s application to this specific group remained limited at that time. More recently, however, this gap in the literature has been addressed: Eke et al. (2024) provided robust evidence in a larger sample (*N* = 484), where the CPORT successfully predicted CSAM-specific recidivism (AUC = 0.73, 95% CI = 0.63, 0.76). This suggests that the initial lack of significance resulted primarily from the low power of the study rather than from a failure of the instrument itself. Consequently, the CPORT now appears to be a promising starting point for determining risk levels in a voluntary prevention context.

The aforementioned study by von Franqué et al. (2023) also investigated the extent to which professionals use the CPORT and other risk assessment instruments with individuals who had a history of CSAM use but presented voluntarily—without a court order—to the DNO prevention project. In contrast to most studies in the field, the criterion used was the self-reported viewing of CSAM during the study period (and not CSAM recidivism determined by information from the criminal record). The criterion could be predicted with the CPORT (AUC = 0.63, 95% CI = 0.53, 0.73), but only when the scale Correlates of Admission of Sexual Interest in Children (CASIC; Seto and Eke, 2017) was used in combination with the CPORT. According to the authors, CPORT and CASIC proved to be promising for the risk assessment of the corresponding sample, with CASIC in particular being held responsible for the predictive validity. However, the authors considered the predictive validity as poor and therefore concluded a need for improvement. The performance discrepancy of this instrument in a non-forensic setting versus a forensic context remains unclear. Preliminary data suggest that non-forensic help-seekers differ significantly from a forensic sample (von Franqué and Briken, 2021) upon which the CPORT was originally validated. In this context, von Franqué et al. (2023) noted that undetected CSAM use and the CASIC emerged as promising specific risk factors on a descriptive level, but without further statistical testing.

Utilizing a subsample from von Franqué et al. (2023), the present study seeks to identify variables that could enhance risk assessment for individuals with a history of CSAM within voluntary treatment settings. This exploratory approach is intended to provide a foundation for developing an instrument tailored to this specific population, aiming to offer incremental predictive validity over the CPORT with CASIC ratings. In addition to CPORT and CASIC items, we incorporate:

- Items from the STATIC-C^2^, an instrument for the risk assessment of CSAM and sexual violence against children (SVC) in a voluntary treatment setting (Briken et al., 2017; Kalt, 2014; von Franqué and Briken, 2021).
- Demographic variables routinely collected as part of network-wide diagnostics and often considered as protective or risk factors for sexual recidivism or CSAM in particular:
  ○ Age: Younger age is consistently associated with higher rates of sexual recidivism (Hanson, 2001; Thornton, 2006) and is thus a core component of various risk assessment instruments (Douglas and Otto, 2020). Specifically, younger age has been linked to increased recidivism risk for CSAM use (Seto, 2025).
  ○ Education: Higher education has been suggested as a protective factor against sexual recidivism in youth (Blum and Ireland, 2004; Stouthamer-Loeber et al., 2004). Additionally, research indicates reduced general recidivism rates among individuals who participated in post-secondary education programs while incarcerated (Batiuk et al., 2005; Chappell, 2004). This is supported by a Google Trends analysis indicating that searches for child-related sexual material are negatively associated with regional education indicators (Walker et al., 2016).
  ○ Employment: Stable employment is associated with desistance from sexual offending (e.g., Lasher and McGrath, 2017) and is consequently integrated into assessments of protective factors for both general violence (de Vogel et al., 2007) and sexual violence (Willis et al., 2022).
  ○ Social isolation: Current partnership status and living situation (living alone) serve as proxy variables for social isolation, which has been identified as a potential risk factor for CSAM users (Seto, 2025). Both aspects are incorporated into the STABLE-2007 (Hanson and Harris, 2007), an instrument that has demonstrated predictive validity for recidivism among CSAM users (Babchishin et al., 2023).
  ○ Parental status: Having children relates to situational contact with minors, which is considered an acute dynamic risk factor for sexual recidivism within the ACUTE-2007 framework (Hanson and Harris, 2007). While the ACUTE-2007 has demonstrated predictive validity for recidivism among CSAM users (Babchishin et al., 2023), the specific item “victim access” (i.e., contact with children in this context) was only a marginally non-significant predictor in that study (HR = 1.91, 95% CI = 0.99, 3.71, *p* = 0.055 for any CSAM use). Conversely, having children could also be interpreted as a component of stable social ties that may counteract social isolation. Given these competing theoretical implications, we decided to examine this variable more closely.
- Previously undetected CSAM use, a variable that emerged as a promising descriptive predictor in a preliminary study (von Franqué et al., 2023) but required rigorous statistical validation.

Accordingly, we address the following research question: Which variables from the item pool seem promising for an optimized prediction of CSAM re-use (i.e., recidivism) in a voluntary treatment setting?

## 2 Materials and methods

### 2.1 Background

The DNO network provides integrative treatment for individuals who:

1. Fear committing SVC and/or using CSAM or
2. Have committed SVC and/or used CSAM in the past, but wish to discontinue this behavior, and are not known to the authorities or
3. Have a conviction of SVC and/or CSAM, but have completed all legal matters, and continue to have treatment needs.

With the network being financed by the joint association of health insurance companies in Germany, the presence of a pedophilic disorder was added as an additional criterion from 2018 onward. According to ICD-10, a pedophilic disorder is defined as sexual impulses and fantasies that relate to children mostly in prepuberty (pedophilia) or early puberty (hebephilia), persist for a period of at least six months, and are associated with clinically significant distress or problematic sexual behavior (World Health, and Organization, 2008). The network currently comprises 13 different sites throughout Germany, including Hamburg and Bamberg (Bavaria). What the network offers is only possible under the special conditions of the German legal system (Briken et al., 2019): the interaction of criminal law, professional secrecy and protective regulations in Germany provides legal certainty for the implementation of the prevention project. Therapists must maintain confidentiality and are not allowed to disclose any information about past sexual abuse or possession of CSAM by their clients, unless there is an acute risk to others. Readers find further information about the network on its homepage at www.kein-taeter-werden.de.

### 2.2 Measures and items

#### 2.2.1 CPORT

The *Child Pornography Offender Risk Tool* (CPORT; Eke and Seto, 2016; Seto and Eke, 2015) is an instrument for measuring the static risk of any sexual recidivism (i.e., sexual contact offenses as well as CSAM and exhibitionist offenses) among adult males with a conviction for possession of CSAM (Seto and Eke, 2015). The instrument consists of seven binary items, which are coded 0 (= not present) or 1 (= present). The instrument covers whether the person to be assessed (1) was younger than 36 years of age at the time of the index investigation, (2) has a criminal record, (3) was conspicuous during early release or parole, (4) has a history of sexual contact offenses, (5) is aroused by prepubescent or early pubescent bodies, i.e., a sexual interest in prepubescent or pubescent children, (6) has possessed more child pornographic content with boys than with girls and (7) whether more media content (i.e., nude images and depictions of partially or fully clothed children that do not meet the legal definition of child pornography) was found with boys than with girls (see Table 2). Accordingly, the total score of the instrument can vary between 0 and 7. According to the manual, scores of 0 and 1 correspond to a low risk, scores of 2 and 3 to a low to moderate risk, a score of 4 to a moderate to high risk and a score of 5 and above to a high risk (Eke and Seto, 2016). The CPORT could not be used in the present study in the way described in the corresponding manual: for example, it was not possible to use official documents such as a criminal record for the assessments. As already described, the CPORT was developed for people with a conviction for CSAM, which only applies to 16% of our sample (3.8% of all subjects had been convicted of both SVC and CSAM).

#### 2.2.2 CASIC

If there is no clinical diagnosis of pedophilic disorder, the fifth CPORT item (admission or diagnosis of sexual interest in children) can be assessed using the *Correlates of Admission of Sexual Interest in Children* (CASIC) scale. However, the instrument was not included in the present study for the diagnosis of pedophilic disorder, but rather because of its presumed relevance for predictive validity in a preliminary study (von Franqué et al., 2023). The CASIC consists of six items that are coded 0 (= no) or 1 (= yes). It assesses whether someone (1) has never been married, (2) has possessed child pornography material or (3) child pornography texts, (4) has had an interest in child pornography for at least 2 years, (5) is or has been involved in a voluntary activity with close contact to children and (6) has had sexual chats with minors. This can result in a total score ranging from 0 to 6. A score of greater than 2 is interpreted as an indication of pedophilic interests. In the original (training) sample, the CASIC total score was significantly associated with the admission of a sexual interest in children (AUC = 0.71; 95% CI = 0.65, 0.77). In the cross-validation sample, an AUC of 0.81 (95% CI = 0.68, 0.95; Seto and Eke, 2017) was obtained. The higher AUC in the cross-validation sample is unexpected and may be due to the small sample size (*N* = 60).

#### 2.2.3 STATIC-C

The STATIC-C (Briken et al., 2017; von Franqué and Briken, 2012) is a tool developed in the context of the DNO network to assess the static risk for SVC or CSAM in a voluntary context. The instrument can be used if only self-reports by the person to be assessed are available and further information (e.g., criminal record) is missing. The STATIC-C was developed on the basis of the STATIC-99 (Hanson and Thornton, 1999). The instrument comprises nine binary items that are coded with 0 or 1. Three further items are coded on scales of 0–2 or 0–3. The items are summarized in Table 2. This results in a total score ranging from 0 to 16. Scores between 0 and 2 are interpreted as low risk of relapse, scores between 3 and 4 as low to medium risk of relapse, scores between 5 and 6 as medium to high risk of relapse, and scores of 7 and more as high risk of relapse.

The psychometric properties of the STATIC-C were evaluated by Kalt (2014), yielding item-level reliability coefficients (Cohen’s Kappa) between 0.56 and 1.0, and an excellent total score reliability (ICC = 0.96). Regarding predictive validity, the instrument achieved an AUC of 0.74 for sexual recidivism over a mean observation period of approximately 38 months. Although this reflects a substantial effect size, the result did not reach statistical significance (*p* > 0.05), likely due to limited statistical power resulting from both the small sample size and the low recidivism rate (3.8%). Additionally, von Franqué and Briken (2021) utilized the STATIC-C to differentiate static risk profiles between a forensic and a non-forensic sample.

#### 2.2.4 Socio-demographic items and undetected use of CSAM

Further items potentially suitable for predicting CSAM re-use (see section “1 Introduction”) were either collected directly during the diagnostic process (age, education, current employment, current partnership, living alone, parenthood/children) or coded on the basis of the case information. Age and partnership are already included as categorical variables in the CPORT/CASIC. Education, current employment, living alone and parenthood/children were coded binary (1 = yes, 0 = no). In addition, undetected use of CSAM was included and coded binary (0 = detected use/prior conviction, 1 = undetected use of CSAM).

### 2.3 Sample

The present study constitutes a secondary analysis of a previously utilized dataset. The original sample comprised 165 individuals, with 80 recruited from the Bamberg site and 85 from the Hamburg site of the DNO network. The preliminary study addressed recidivism risks for SVC and CSAM among a non-forensic sample (for further details, see von Franqué et al., 2023, p. 7).

For the purpose of the current study, participants were included based on the following criteria:

1. They were male (while the DNO network generally includes female participants, they were excluded from this analysis to ensure sample homogeneity and to control for gender-based variance), and
2. They had a confirmed history of CSAM use. The latter criterion was applied because the study focuses specifically on CSAM recidivism rather than its initial onset.

Applying these inclusion criteria resulted in a final sample of 131 men (72 from Hamburg and 59 from Bamberg). Detailed descriptive statistics of the sample are provided in section “3 Results.”

### 2.4 Procedure

#### 2.4.1 Diagnostic phase

Participants underwent semi-structured clinical interviews conducted by trained psychologists or psychiatrists. To mitigate participant fatigue, the interview duration and format were adjusted by site: in Hamburg, interviews were split into 1-h sessions over several days, while in Bamberg a single session with a 2-h limit was implemented and only if necessary, further appointments for diagnostics were arranged. The interview protocol focused on socio-demographic and biographical data, mental health history, and sexual development. Crucially, it detailed both detected (official charges or convictions) and non-detected (unrecorded) histories of SVC and CSAM. This information was supplemented by standardized self-report questionnaires assessing criminogenic attitudes, hypersexuality, impulsivity, anxiety, and depression. Due to confidentiality concerns and the participants’ fear of legal repercussions or stigmatization, external sources (e.g., criminal records or prior treatment reports) were generally not accessible.

The data collected through this comprehensive process served as the basis for ICD-10 diagnostic coding and STATIC-C ratings. Furthermore, the CPORT and CASIC were completed retrospectively based on these detailed case files, as they were not part of the original clinical diagnostic routine. Great care was taken to ensure that only initial information was used to assess the CPORT and CASIC, not follow-up information. Five professionals from two different sites assessed the participants.

#### 2.4.2 Evaluation phase

At the end of the diagnostic phase, the participants took part in psychotherapeutic treatment and/or follow-up interviews. In this study, we refer to this period as the evaluation phase, during which we examine the respective file for the re-use of CSAM. The participants’ files were evaluated by five professionals for the occurrence of CSAM based on session protocols, session questionnaires and annual risk assessments. The average evaluation period was 2.5 years.

#### 2.4.3 Criteria

For the purpose of the present study, CSAM was defined as photorealistic material (including AI-generated media) in which at least one child (1) is depicted in an unnatural, sexualized manner, (2) performs acts on themselves that are understood to be sexual, or (3) is involved in attempted, actual, or threatened sexual acts with at least one other person. Based on a commonly used definition (“usually defined as a new sexual arrest, charge, or conviction,” Seto and Eke, 2015, p.416), recidivism in this study is operationalized as the self-reported re-use of CSAM following the established reference point (i.e., after the end of the diagnostic phase/the onset of the evaluation phase). This definition accounts for both continuous and intermittent patterns of CSAM re-use. To maintain a specific focus on CSAM-related behavior, other forms of problematic behaviors, such as sexual or non-sexual violence, were not classified as recidivism in this context.

Five professionals across two sites conducted the file assessments. In Hamburg, a Fleiss’ Kappa coefficient of 0.72 was obtained for three raters based on a random sample of 24 out of 85 participants. In Bamberg, a Cohen’s Kappa coefficient of 0.73 was achieved based on a random sample of ten participants and two raters. According to Landis and Koch (1977), these coefficients indicate “substantial agreement.” These results may be attributed to the extensive nature of the files used for assessment, as CSAM-related information was often documented across multiple sources (e.g., session protocols or questionnaires). Discrepancies in ratings likely arose when specific information within these comprehensive records was overlooked by individual raters.

### 2.4.4 Design

The present study primarily uses a retrospective cohort design. The CPORT was assessed based on case information available at the end of the diagnostic phase. In contrast, the STATIC-C data were already part of the diagnostic phase. The occurrence of CSAM was assessed using all available information during the evaluation phase.

### 2.4.5 Statistical analysis

The evaluation and selection of items follows the external criterion-related strategy of scale construction (Hock et al., 2023, Chapter 11.2.4). In this strategy, which has a long tradition in psychological assessment (Burisch, 1984), items are compiled into a scale according to their validity for a criterion (e.g., Garb et al., 2011). Sometimes the bivariate correlations between item and criterion are used for this purpose (e.g., Elleman et al., 2020). However, this is not optimal because good items must show *incremental* validity, which is not tested with the bivariate correlation. We therefore used a procedure that selects items with regard to their incremental validity.

Because the aim of the current study was to develop an index scale consisting of a simple sum score, the item selection was carried out in such a way that the validity of the sum score was optimized. For this purpose, items that maximized the incremental validity of the resulting sum score were successively selected from the items used to assess the risk of relapse. In the first step of the procedure, the item with the strongest association with the criterion (CSAM recidivism) was selected. In the second step, the item was selected for which the sum of the already selected item and the new item achieved the maximum validity. This process was continued as long as the validity of the sum score could be increased by adding new items. The procedure is thus similar to forward selection in stepwise multiple regression.

Items that did not show a positive bivariate correlation with the criterion (after keying the item in the direction of the criterion) were removed from the item set in advance. This made sense, as clear assumptions about the direction of the correlation were available for all items. The area under the receiver operating characteristic curve (AUC) resulting for the sum score as a predictor was selected as the association measure to be optimized.

To prevent overestimation of the validity of the sum score by the selection procedure, the procedure was cross-validated. We used Monte Carlo cross-validation (MCCV; also known as repeated random sub-sampling), which is considered more reliable than competing methods (Xu and Liang, 2001). MCCV is associated with a relatively high computational effort, but this is not significant for small samples such as the present one.

In MCCV, the sample is repeatedly split into two non-overlapping parts, resulting in two data sets. One is used to “train” the model, in this case to select suitable items for the sum score, while the other is used to validate the selection. As the validation data set represents a new data set, overestimates of performance due to adjustments of the model to the specifics of the training sample are avoided. This provides a realistic assessment of its performance when used in new samples. The repetition of the procedure results in a distribution of validity coefficients, which makes it possible to assess the expected performance variance of a procedure in future studies. Distributions are also obtained for the validity estimates from the training sample and for the number of model parameters (in this case, the number of items selected). The difference between the validity in the training sample and that in the validation sample provides a measure of the overestimation of validity that would occur if only the original sample were used for its evaluation. We drew 1,000 random samples by stratified sampling, in which the groups of interest (no relapse, relapse) are represented as equally as possible in the training and validation data sets.

The advantage of this procedure over the stepwise regression procedures is that the validity of the index scale (the sum score) to be used later is tested directly. In contrast, with regressions a linear combination with differently weighted items was tested. Experience in test construction shows that the superiority of weighted predictors, which is actually to be expected, does not always manifest itself in new samples (Dawes, 1979; Elleman et al., 2020; Wainer, 1978), so that unweighted sum scores are preferred for reasons of simplicity. For the same reason, unweighted item scores are usually used in tests to measure latent traits instead of scores that are weighted according to factor loadings or item discriminations.

To compare our approach with a well-established procedure, we also used stepwise logistic regression. In contrast to the simple selection procedure used here, in the regression items are weighted with respect to their contribution to the prediction of the criterion. The item selection was carried out both forwards (inclusion of items that contribute to the prediction) and backwards (removal of items that contribute nothing or little) in such a way that the Akaike information criterion (AIC) was minimized. This should achieve a compromise between prediction performance and simplicity of the resulting model. The process was terminated if no further reduction of the AIC was achieved. An MCCV was also performed for this selection, again with 1,000 sample draws.

The analyses were performed with R (R Core Team, 2024). The described forms of item selection and cross-validation are implemented in the R package *simpredsel* (Hock, 2024). For the stepwise logistic regression the package relies on the *step*-Function implemented in the package MASS (Venables and Ripley, 2007). It should be noted that the division of the sample as part of the cross-validation serves to determine the validity of the selection procedure, not to determine the best items. The final item selection was made on the basis of the total sample, as this provides a broader information base than the subsamples used for cross-validation.

## 3 Results

### 3.1 Socio-demographic items and non-detected use of CSAM

Table 1 shows the distributions of the variables described and their association (Pearson correlation or Phi coefficients) with CSAM recidivism. As all participants were male, gender is not specifically listed here. Younger people, single people, and those without having children of one’s own had significantly higher rates of recidivism. Conversely, older age, being in a partnership, and parenthood were identified as protective factors. Additionally, there was a further association with undetected CSAM use (conversely, 16% of our participants had previously been detected, meaning they had been convicted for using CSAM) which was associated with an increased rate of CSAM recidivism. In contrast, the duration of education, an existing employment relationship and living alone were not predictive.

**TABLE 1:** Socio-demographic items and undetected use of child sexual abuse material (CSAM): distribution and correlation with CSAM recidivism.

Variable	*M* (SD) or percentage	*r*
Age (years)	35.87 (11.53)	−0.19*
Duration of education/school > 10 years	70%	0.02
Current employment status	73%	−0.04
Partnership	50%	−0.21*
Living alone	44%	0.14
Parenting/children	30%	−0.29*
Previously undetected CSAM use	84%	0.23*

*r* = correlation with CSAM recidivism. *N* = 131, of which *n* = 52 (39.7%) relapsed. *P < 0.05 (one-tailed).

Age and partnership are also addressed in the instruments used here (STATIC-C, CPORT/CASIC), whereas parenthood and undetected use of CSAM are not. As previously described in the introduction, both might represent good candidates for a new scale for estimating the risk of recidivism. Therefore, we included them in the item selection procedure. Parenthood was inverted for this purpose (having children of one’s own = 0, no children = 1) so that all items were coded in the same direction.

### 3.2 Items

The items of the instruments used are summarized in Table 2. There are 25 items in total. As already mentioned, most items were binary. Items were coded as 1 if the attribute was present and 0 if it was absent. For items containing more than two response categories, higher scores could be achieved, which is also indicated in Table 2. Table 2 also shows the mean values of the items (i.e., the relative frequencies for the binary items) and the correlations with CSAM recidivism (for binary items, the correlations correspond to Phi coefficients).

**TABLE 2:** Mean and correlation with child sexual abuse material (CSAM) recidivism of items used.

Instruments	Item no.	Item	*M*	*r*
CPORT	1	Age ≤ 35 years	0.54	0.21*
CPORT	2	Any prior criminal history	0.27	−0.08
CPORT	3	Any failure on conditional release	0.01	−0.07
CPORT	4	Any prior or index contact sexual offense history	0.05	−0.05
CPORT	5	Pedophilic or hebephilic interests, clinical diagnosis	0.96	0.08
CPORT	6	More boy than girl child pornography content	0.21	0.13
CPORT	7	More boy than girl other child-related content (cf. CPORT 6)	0.21	0.13
CASIC	1	Never married	0.44	0.30*
CASIC	2	Child pornography movies	0.57	0.26*
CASIC	3	Child sex stories	0.46	0.01
CASIC	4	Interest in child pornography spanned 2 or more years	0.70	0.32*
CASIC	5	Volunteers with children	0.15	0.05
CASIC	6	Online sexual communication with a child	0.15	−0.08
STATIC-C	1	Aged 18–24.99 (cf. CPORT 1)	0.19	0.04
STATIC-C	2	Never lived with lover for at least two years (cf. CASIC 1)	0.44	0.33*
STATIC-C	3	Diagnosis of personality disorder	0.15	0.11
STATIC-C	4	Previous convictions (see CPORT 2)	0.22	−0.13
STATIC-C	5	Non-sexual violent crimes	0.06	−0.08
STATIC-C	6	Diagnosis of pedophilic disorder (no = 0, yes = 1, exclusive type = 2)	1.11	0.04
STATIC-C	7	Diagnosis of other paraphilic disorder (no = 0, yes = 1, sadism = 2)	0.18	0.02
STATIC-C	8	Ever used child sexual abuse material	1.00	–
STATIC-C	9	Number of sexual violence victims (0, 1 = 0, 2 = 1, 3 = 2, 4 + = 3)	0.49	−0.05
STATIC-C	10	Any unrelated sexual violence victim(s)	0.25	0.00
STATIC-C	11	Any stranger sexual violence victim(s) (24-h rule)	0.08	−0.12
STATIC-C	12	Any male sexual violence victim(s)	0.12	0.03

*r* = correlation with CSAM recidivism. *N* = 131. *p < 0.05 (one-tailed). The term “child pornography” is problematic because it can mistakenly suggest consent. Nevertheless, the term is used here in order to correctly cite the items of instruments used.

**FIGURE 1:**
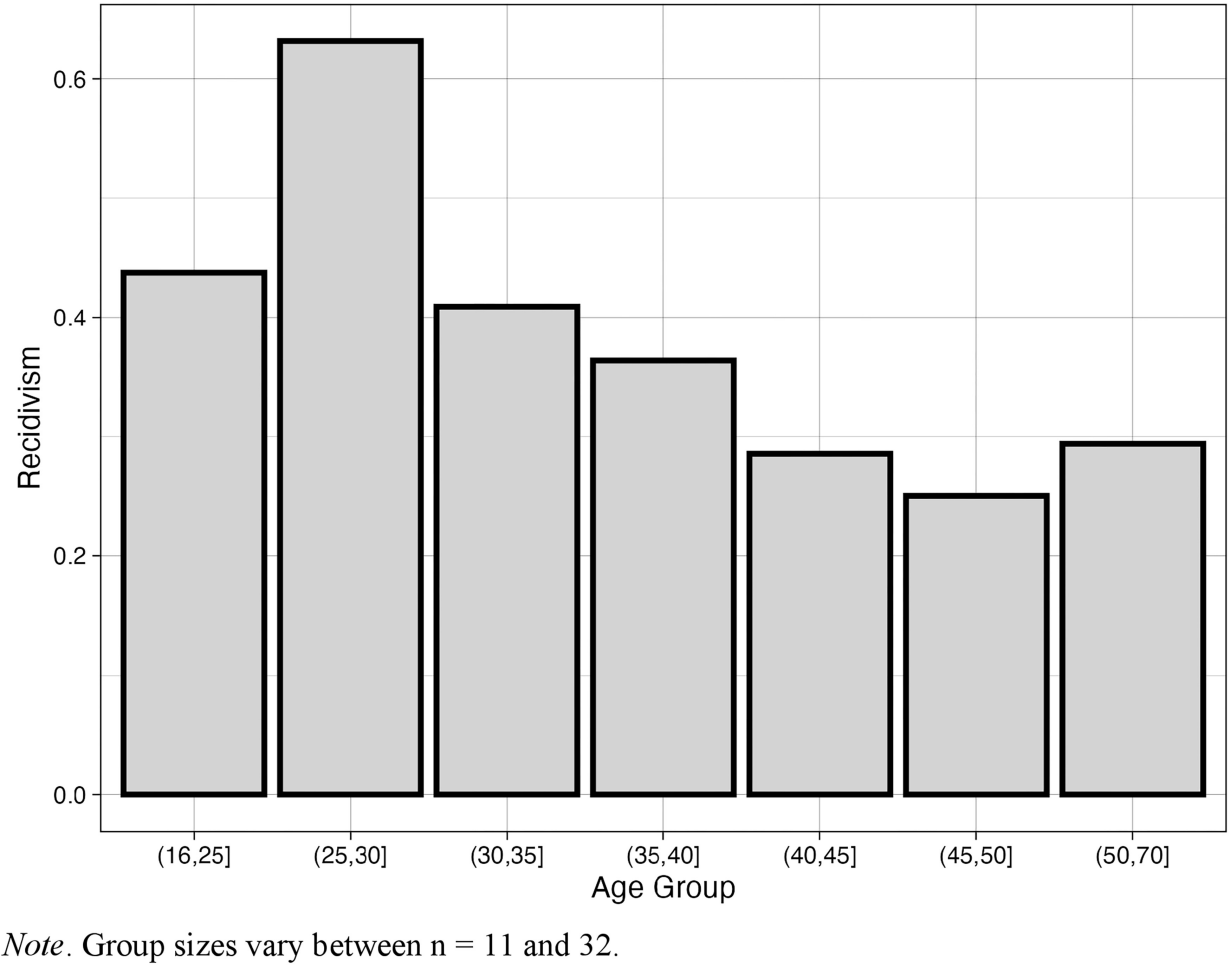
Risk of relapse as a function of age.

Some items occur in identical or very similar form in several instruments, which is indicated in Table 2 for the items concerned. For item selection, multiple uses of items must be excluded. We chose the variant that is listed first in the table. An example of this is age, which is represented with different cut-off values in CPORT and STATIC-C. Here we opted for the CPORT version, in which people aged 35 or younger receive a score of 1, while older people receive a score of 0. The correlation with recidivism (Table 2) and an inspection of the distribution of recidivism risk by age (Figure 1) showed that a cut-off value of 35 years discriminates somewhat better between recidivists and non-recidivists than the cut-off value of 25 years used by the STATIC-C. CPORT item 7 and the STATIC-C items 2 and 4 (see Table 2) were excluded from the item selection, as these correlate with other items (CPORT item 6 or CASIC item 2 and CPORT item 2) by almost 1 and thus provide practically the same information.

Table 2 shows that some items occurred so rarely or so frequently in the current sample that they can only be slightly associated with the risk of relapse from the outset. Due to the selection of the sample, there is no variation in the STATIC-C item 8 (*ever used CSAM*). This item was therefore excluded from the selection procedures. Very rare (occurrence ≤ 5%) are CPORT items 3 (*failure of probation and similar*) and 4 (*convicted hands-on sexual offense*). Very common (occurrence ≥ 95%) is CPORT item 5 (*pedophilic or hebephilic sexual interests, clinical diagnosis*). Although these will contribute little or nothing to validity in the present study, they could be important in samples with greater heterogeneity of the corresponding characteristics.

Significant correlations with recidivism risk were found for five items: CPORT 1 (*age* ≤ *35 years*), CASIC 1 (*never married*), CASIC 2 (*possessed child pornography videos*), CASIC 4 (*interest in child pornography spanned 2 or more years*) and STATIC-C 2 (*never lived with lover for at least two years*). The corresponding correlations varied between *r* = 0.21 and 0.33.

### 3.3 Measures

In order to gain a basis for comparing the validity of the newly selected items, we first calculated the validity of the individual measures used (see Table 3). CASIC achieved a substantially higher validity (AUC = 0.68) than the other instruments (AUC = 0.55 and 0.56) and was the only instrument whose validity was statistically significant.

**TABLE 3:** Distribution parameters and predictive validities of the instruments used.

Instrument	*M* (SD)	AUC	95% CI
CPORT with clinical diagnosis	2.25 (1.10)	0.56	0.47–0.66
CASIC	2.47 (1.27)	0.68	0.59–0.77
STATIC-C	4.29 (2.16)	0.55	0.45–0.65

AUC = area under the ROC curve; CI = confidence interval.

### 3.4 Item selection

Item selection using stepwise logistic regression yielded a mean AUC of 0.86 (SD = 0.05) for the training data and an AUC of 0.73 (SD = 0.05) for the validation data. The latter value, which is decisive for our purpose, is thus higher than the validity of CASIC alone. On average, k = 7.18 (SD = 2.50) items were selected. However, in the final model, which was calculated on the entire data set, only the demographic variable *no children*, *the previous, undetected use of CSAM* and the items CASIC 1 (*never married*) and CASIC 4 (*interest in child pornography spanned 2 or more years*) were represented with a positive weight.

The selection with regard to the optimization of the sum score showed a mean AUC of 0.84 (SD = 0.03) for the training data. For the validation data, the AUC was 0.73 (SD = 0.05). On average, k = 6.68 predictors were selected (SD = 1.63). The final model contained six predictors. These included the four already mentioned items and additionally the items CPORT 5 (*pedophilic or hebephilic interests, clinical diagnosis*) and CASIC 2 (*possessed child pornography videos*).

Table 4 summarizes the demographic variables and items relevant for the prediction of CSAM recidivism according to the different methods used (bivariate correlation, stepwise regression, optimization of the validity of the sum score).

**TABLE 4:** Items for predicting child sexual abuse material (CSAM) relapse, differentiated by selection methods.

Variable	Bivariate	Re-gression	Total score
Demographic variables			
Age (years)	*	—	—
Current partnership	*	—	—
Parenthood/no children	*	*	*
Previously undetected CSAM use	*	*	*
Items of different instruments			
CPORT 1, age ≤ 35 years	*	–	–
CPORT 5, pedophilic or hebephilic interests, clinical diagnosis	–	–	*
CASIC 1, never married	*	*	*
CASIC 2, possessed child pornography videos	*	–	*
CASIC 4, interest in child pornography spanned 2 or more years	*	*	*
STATIC-C 2, never lived with lover for at least 2 years (cf. CASIC 1)	*	–	–

Bivariate = significant bivariate correlation. Regression = selection in stepwise multiple regression. Total score = optimization of the sum score. Items marked with * were selected using the corresponding method. Variables marked with “—” were not included in the item selection procedures. The term “child pornography” is problematic because it can mistakenly suggest consent. Nevertheless, the term is used here in order to correctly cite the items of established instruments.

### 3.5 Score distribution and risk of relapse

**FIGURE 2:**
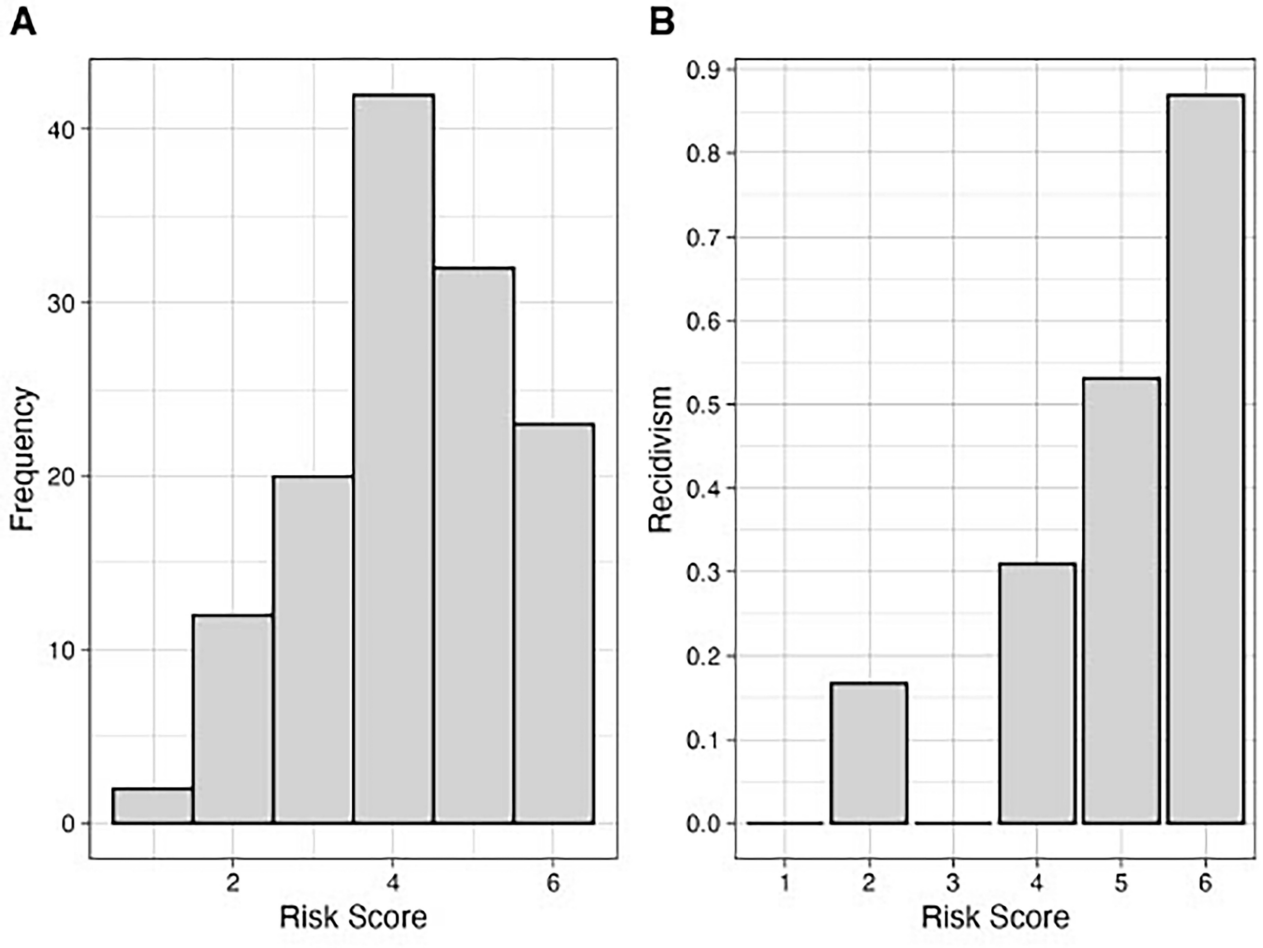
Distribution of risk scores **(A)** and risk of recidivism among score group **(B)**.

Figure 2 shows the distribution of the risk score resulting from the sum score optimization (panel A) and the risk of recidivism as a function of the sum score of the new scale (panel B). The distribution of the sum score in Figure 2 shows that the majority of people have a high-risk score (≥4). However, the risk of relapse was substantially higher for people with a risk score of greater than or equal to 5 than for people with a lower sum score. Values of 0 did not occur, while the highest possible value of 6 points was assigned 23 times (17.6%).

Hanson et al. (2017) propose a five-level system for classifying the risk of relapse, which includes statistical criteria and psychological aspects. If only the statistical criteria are used, the levels are defined by the following percentile rank ranges: Level I: <5%, Level II: 5%–29%, Level III: 30%–49%, Level IV: 50%–84%, Level V: >84%. This results in the allocations shown in Table 5 for the available data.

**TABLE 5:** Risk levels according to Hanson et al. (2017).

Risk score	Level according to Hanson et al. (2017)	*n*	Recidivists	Recidivists %
1	I	2	0	0
2–3	II	32	2	6.2
4	III	42	13	31.0
5	IV	32	17	53.1
6	V	23	20	87.0

*n* = number of persons in the stage; recidivists (%) = number and relative frequency of recidivists in the stage, see Figure 2.

## 4 Discussion

The aim of the present study was to identify variables that could be promising for the future development of a risk assessment tool for CSAM recidivism in people under voluntary treatment. First, in addition to previous findings, we found that it is apparently the CASIC that is responsible for the predictive validity of the combined assessment with CPORT and CASIC, as the CPORT with clinical pedophilia diagnosis alone did not significantly predict CSAM recidivism (von Franqué et al., 2023). It is worth noting that the CASIC is not an instrument genuinely developed for risk assessment, but rather a tool for assessing pedophilic or hebephilic interests. However, the findings do not reveal which items of the CASIC contribute to the predictive performance. To clarify this point, we conducted item-level analyses.

In total, we identified six items as promising candidates for a risk assessment instrument: the items *no children* and *Previously undetected use of CSAM*, the CASIC items CASIC 1 (*never married*), CASIC 4 (*interest in child pornography spanned 2 or more years*) and CASIC 2 (*possessed child pornographic videos*) as well as the CPORT item CPORT 5 (*pedophilic or hebephilic interests, clinical diagnosis*). As the overview in Table 4 makes clear, not all variables that prove to be significant in bivariate analyses are also incrementally valid. This applies to age (CPORT 1) and the absence of a long-term relationship (STATIC-C 2). The commonalities of these items with the others are apparently too large to improve the validity of the prediction. The fact that age under 36 years (CPORT 1) is not a predictor is probably due to the fact that this item is highly correlated with *parenthood* (*r* = 0.47), *never married* (CASIC 1; *r* = 0.41) or *never lived with lover for at least two years* (STATIC-C 2, *r* = 0.44; all *p*_*s*_ < 0.001). In general, the age criterion should be re-examined, also with regard to the optimal cut-off value. In any case, age is not a causally relevant variable for relapse *per se*, but a placeholder for psychological processes that are linked to age.

In line with the general finding that past offending is a robust predictor of future delinquency (e.g., Bonta and Andrews, 2023; Hanson and Morton-Bourgon, 2005; Seto, 2025), our procedure selected three items that characterize prior CSAM use. These findings support the view that CSAM recidivism risk is associated with a pattern of previous CSAM usage (Napier et al., 2024). The items CASIC 2 (*possessed child pornography videos*), CASIC 4 (*interest in child pornography spanned 2 or more years*), and the item *previously undetected use of CSAM* proved to be relevant. The first two items specify aspects of the quality (namely videos instead of pure image material) and quantity (namely the period of use) of past behavior and thus make it clear that past use should be differentiated. The selection of the third item confirms the assumption that the undetected use of CSAM in the past can be important for predicting CSAM re-use (von Franqué et al., 2023). To our knowledge, this predictor has not yet been considered. Most prediction tools (cf. Douglas and Otto, 2020) only use convictions or charges to assess risk. This indicates that our final scale is better at predicting whether someone reused CSAM than it was before undetected use was added. In future studies incorporating more participants it would be interesting to contrast undetected use of CSAM and convictions with respect to their predictive power. It seems possible that in such an extended sample both are needed for an optimal prediction of CSAM re-use. In any case, traditional assessment tools relying solely on official criminal records (convictions) may underestimate this risk as the “undetected” status is overlooked. The question arises as to how our results based on self-reports should be interpreted. From a critical point of view, these could simply (and trivially) reflect the fact that people who admit to past usage behavior were also willing to provide corresponding information about future usage behavior. However, this interpretation is not consistent, as all subjects in our sample reported usage behavior in the past, but the selected items differentiated between subjects with and without CSAM re-use. In addition, the hypothesis that the results are due to a specific subgroup of subjects who, unlike the other participants, reported truthfully about the specifics of their problem behavior cannot sufficiently explain the results. Other offense-related aspects (e.g., CASIC 3 *possessed child pornography histories* or CPORT 6 *dominance of boys in child pornography*) did not differentiate between recidivism and lack of recidivism. We therefore assume that the items are actually risk factors. Of course, these items would have to prove themselves in further studies.

Two further selected items relate to the social situation of the person being assessed, namely the conditions in the partnership and family: The item CASIC 1 (*never married*) proved to be predictive for the re-use of CSAM in our sample. A stable relationship can therefore be seen as a protective factor against relapse with CSAM. Partnerships also play an important role in risk assessment tools for other types of delinquency (cf. Douglas and Otto, 2020). In addition, the meta-analysis by Babchishin et al. (2015) shows that individuals with a history of exclusive CSAM offending are less likely to have stable relationships compared to those with a history of other sexual offenses. It is debatable what causes difficulties in this area. Several hypotheses exist: One might argue that these difficulties are due to deviant sexual arousal (Seto et al., 2021)—specifically, a lack of interest in stable relationships with adults (Barroso et al., 2026; Blanchard and Bogaert, 1997). Alternatively, one might suggest the cause is a lack of emotional regulation (Magaletta et al., 2014; Mann et al., 2010), which makes it difficult to form or maintain intimate relationships (Morgan and Lambie, 2019). In this context, self-esteem issues (Babchishin et al., 2015; Bartels and Merdian, 2016) as well as experiences of loneliness or relationship difficulties (Babchishin et al., 2015; Bartels and Merdian, 2016; Brankley et al., 2021; Quayle and Taylor, 2002) could also serve as explanations. Another possibility is that a stable partnership leads to increased control over online behavior.

The demographic item *No children* was also selected. Consequently, within our sample, parenting may be regarded as a protective factor against CSAM recidivism rather than an acute dynamic risk factor, as the findings of Babchishin et al. (2023) might otherwise suggest. This finding is noteworthy because, to our knowledge, no other risk assessment instrument for sexual delinquency explicitly lists parenthood as a protective factor (cf. Douglas and Otto, 2020), despite the fact that individuals who use CSAM generally tend to have fewer or no biological children (Barroso et al., 2026). However, it could be considered part of the “life goals” item in the Structured Assessment of Protective Factors (SAPROF; de Vogel et al., 2007) and the SAPROF Sex Offender Version (SAPROF-SO; Willis et al., 2022). The exact causal role of parenthood with regard to CSAM recidivism remains unclear. According to an initial hypothesis, parenthood and anticipated reuse of CSAM could trigger cognitive dissonance (Festinger, 2012), which affected individuals then attempt to reduce by refraining from recidivism. From a risk management perspective, however, it must be objected that this would only provide a statement about the risk of CSAM re-use, but nothing about the risk of SVC.

Finally, the clinical item CPORT 5 (*pedophilic or hebephilic interests, clinical diagnosis*) was selected, which showed only a weakly positive (insignificant) bivariate correlation with the criterion. This item was also not included in the stepwise regression. In contrast to regression models, our procedure does not assign “penalties” for model complexity (like the AIC or other information criteria) or insignificance. It is therefore less strict than alternative procedures. However, for the present purpose of selecting potentially suitable items for a new scale, a high sensitivity of the procedure should be seen as an advantage rather than a disadvantage. This should keep the preliminary item pool open for candidates that may later prove to be predictively useful.

Substantively, this result is consistent with a body of findings concerning the characteristics of individuals with a history of CSAM offenses: For example, the people surveyed in a study by Seto et al. (2010) reported that they used CSAM because of their sexual interest in children. Babchishin et al. (2015) showed in a meta-analysis that convicted persons with CSAM have stronger paraphilic—especially pedophilic and hebephilic—interests compared to persons with SVC. Seto et al. (2021) found in their online sample of 1,036 men and women that pedohebephilic interest was significantly associated with concordant behavior among heterosexual men, heterosexual women, and non-heterosexual men. Similarly, Barroso et al. (2026) identified in their systematic literature review that individuals using CSAM frequently report experiencing sexual attraction toward children. However, the results of von Franqué et al. (2023) in combination with the findings of the present study suggest that the predictive validity of the clinical diagnosis alone might be rather low. Thus, CPORT with CASIC rating and even CASIC alone was superior to assessment with CPORT with clinical diagnosis.

Our results align with the motivation-facilitation model (Seto, 2019, 2025), where the paraphilic interest acts as a motivator. However, this motivation is only effective when combined with facilitating factors, such as undetected CSAM use, a lack of a relationship, or no children. Learning experiences (e.g., a long-term interest in CSAM) and a lack of consequences further contribute to CSAM recidivism. This causal hypothesis can be well reconciled with our findings, but needs to be confirmed by further work with a different research design.

Interestingly, our approach primarily selected items that serve as indicators of paraphilic interest (Seto, 2025)—one of the core dimensions of recidivism risk in sexual offending (Hanson and Morton-Bourgon, 2005). In contrast, items associated with the second major risk dimension, antisociality (e.g., CPORT 1, CPORT 2, CPORT 3), were not selected. This discrepancy may explain why established risk assessment tools, which often rely heavily on antisocial history, appear less effective within voluntary prevention samples. In our study, antisociality-related items did not contribute to risk differentiation, likely due to the low prevalence of antisocial traits within the sample. This interpretation is supported by research indicating that individuals who exclusively use CSAM (comprising 89 individuals in our sample) exhibit significantly lower levels of antisociality compared to those who commit contact sexual offenses or mixed offenses (Babchishin et al., 2015; Henshaw et al., 2018).

For the tool developed by our selection process, an overall AUC value resulted that was superior to the AUC value of CASIC alone and the value of CPORT with CASIC reported by von Franqué et al. (2023) in both the training data and the validation data. Thus, we followed our idea to develop a risk assessment instrument for resource allocation optimized for men in voluntary treatment (von Franqué et al., 2023). However, the right-skewed distribution of the sum score in Figure 2 shows that extremely low values (i.e., values of 0) do not occur, while the highest possible value (6) is relatively common. This indicates that the items as a whole are somewhat too easy for the present sample and should be supplemented with more difficult items if possible. The risk of recidivism can be described as very low for scores lower than 4 and relatively high for scores higher than 4. With regard to the general accuracy of the prediction, the cut-off value for a binary decision (relapse, no relapse) would be between 4 and 5 (i.e., a relapse would be predicted from a value of 5). For this cut-off value, the present sample yielded a sensitivity of 0.77 and a specificity of 0.71. However, it should be noted that these values have a positive bias, as they were obtained on the basis of the same sample that was used for item selection.

To allocate treatment resources effectively in accordance with the risk principle (Bonta and Andrews, 2023) for individuals with a history of CSAM use in a preventive context, several implications arise from current findings. In principle, help-seeking individuals could be assessed using the CPORT in combination with the CASIC. This combination demonstrated predictive validity in our preliminary study—based on self-reported data—and thus appears preferable to instruments like the STATIC-C, which is currently employed within the DNO network. Given that 80% of the subjects in the preliminary study had used CSAM, the CPORT and CASIC combination offers a broad range of applications and has also shown predictive validity for sexual violence against children (SVC; von Franqué et al., 2023). However, the results of the present study suggest that the CASIC alone may be even better suited for differentiating between potential clients. Our findings are thus consistent with a recent study in which the total CASIC score served as an indicator of recidivism in a sample of individuals convicted of CSAM offenses (Seiser et al., 2026). While CASIC and CPORT benefit from ongoing research in numerous studies (cf. Seto, 2025), our newly developed scale achieved the highest predictive validity by incorporating specific characteristics of this unique clientele. With reference to Table 5 and the publication by Hanson et al. (2017), treatment planning can be carried out in accordance with the risk principle. Nevertheless, it must be noted that this new measure has not yet been as extensively empirically validated as the CPORT/CASIC. Although our results are encouraging, the items would need to be tested on new samples before clinical application can be considered. In this respect, further research with the scale is necessary. All instruments rely on static risk factors and are therefore of limited value for concrete treatment planning. However, static items such as *Never married* can provide initial indications for intervention planning; for instance, they may suggest a need for enhancing interpersonal or relationship skills. This is consistent with the need principle of the RNR model, which mandates that interventions should address criminogenic needs or dynamic factors directly associated with recidivism (Bonta and Andrews, 2023).

### 4.1 Limitations and future perspective

Despite promising results, our study has some limitations: First, a major limitation is the relatively small sample size, which makes our estimates less precise than those of studies with larger samples. The evaluation period of approx. 2.5 years is also significantly shorter than the follow-up periods used in other studies (cf. Baskurt et al., 2025; Seto et al., 2011). An extension of the evaluation period would probably have led to even higher recidivism rates of CSAM. In this respect, it would make sense to replicate our results after a few years and with a larger number of people, recruited, e.g., via other sites of the DNO network. In addition, our sample was restricted to individuals with a confirmed history of CSAM use. While this focus was necessary to evaluate recidivism-based risk, it limits the generalizability of our findings to the broader population of the DNO prevention network. Specifically, our analysis does not account for individuals who seek help due to a perceived risk of initial CSAM use but have not yet engaged in such behavior. Future research should investigate whether the predictors identified here differ for individuals at risk of initial onset versus those at risk of recidivism. Furthermore, the reliance on a help-seeking, non-forensic sample may introduce a selection bias, as these individuals might possess higher levels of motivation or different risk profiles compared to a forensic clientele.

Our definition of CSAM is very strictly based on the German criminal justice system and its definition of so-called child pornography. We have not taken into account material depicting sexual acts by, on or in front of a person aged between fourteen and seventeen, which is referred to as youth pornography in the German Criminal Code. In contrast, international work includes any material that depicts sexual activities involving persons under the age of 18 (International Centre for Missing and Exploited Children, 2023; Seto, 2025). It is possible that a different definition of the criterion would have led to different results. In addition, our definition of CSAM recidivism is subject to criticism as it confounds continuous and intermittent patterns of CSAM re-use. These may represent two distinct behavioral groups influenced by different sets of predictors. Future research involving larger samples should address this by implementing specific waiting periods after the reference point to differentiate between these trajectories. Such a distinction would allow for a more nuanced understanding of whether stable, persistent use and episodic, recurrent use are driven by the same or divergent risk factors.

A primary limitation of this study is that all results are based on participants’ self-reports. While using self-reports as the sole criterion deviates from standard forensic practice, this approach is not without precedent in the literature (e.g., Monahan et al., 2001). Although the risk assessment instruments, including the CPORT, were scored based on detailed clinical interviews, official criminal records—typically a prerequisite for manual-compliant scoring—were unavailable. This reliance on a single data source may have influenced the results in two ways: first, through a potential underreporting of past CSAM use or risk behaviors due to social desirability bias; and second, by reducing the reliability of items that participants might intentionally minimize in the absence of objective file data. Despite these constraints, self-reports often remain a practical necessity in non-forensic, voluntary prevention contexts where anonymity and therapeutic trust are paramount.

It is possible that participants provided valid information specifically because of the protections afforded by medical confidentiality (Briken et al., 2019). Consequently, the newly identified factors—such as undetected CSAM use or parenthood—might not generalize to other contexts, such as mandated forensic evaluations. In such cases, the application of these findings may be specific to the DNO network. However, given the network’s significant reach—with nearly 400 patients currently in treatment and approximately 2,000 treated to date—these insights remain highly relevant^3^. Future studies should aim to validate whether these predictive patterns hold true when supplemented by official records in both forensic and non-forensic contexts. Furthermore, integrating self-reports with collateral data would allow for a more robust assessment of the “dark field” phenomenon, accounting for the discrepancy between committed and officially reported problem behavior (cf. Pham et al., 2021; Stoltenborgh et al., 2011)

Finally, the present study relied exclusively on instruments utilizing static risk factors. According to various studies (Bonta and Andrews, 2023; Douglas and Skeem, 2005; Hanson and Harris, 2000; Mann et al., 2010), a comprehensive risk assessment should incorporate dynamic risk factors alongside static ones. Such an integrated approach would more effectively implement the need principle of the RNR model, which focuses on modifiable factors associated with criminogenic characteristics (Bonta and Andrews, 2023). Although the items used here are technically static, some—such as marital status—serve as proxies for modifiable competencies, like relationship skills, that could be prioritized in intervention planning. Future research should therefore further investigate the interplay between static and dynamic factors specifically within voluntary prevention frameworks. This would be in line with newer network approaches (such as the Neurobehavioral Model for Risk of Sexual Reoffending; van den Berg et al., 2023) to risk assessment, according to which recidivism can be understood as a dynamic process that depends on many different variables and their interaction in the network. A promising instrument could be the STABLE-2007 (Hanson and Harris, 2007). According to various findings (Babchishin et al., 2023; Brankley et al., 2021), the tool could be used to validly estimate the risk of CSAM re-use in forensic samples. In the dark field, however, only the relationship between STABLE-2007 (Hanson and Harris, 2007) and lifetime offending has been considered so far (Bergner-Köther et al., 2024). In this respect, further investigations of dynamic risk factors in samples of men with pedophilic disorder and without court orders would be a welcome addition on the way to a comprehensive instrument.

### 4.2 Conclusion

In order to allocate treatment resources appropriately according to the risk principle (Bonta and Andrews, 2023), adequate risk assessment instruments are essential for institutions working with men with pedophilic interest in a non-forensic context. Of the existing tools STATIC-C, CASIC, and CPORT with clinical diagnosis, only CASIC proved to be predictively valid for the CSAM recidivism. According to our results, the items *no children* and *previous, undetected use of CSAM* as well as CASIC 1 (*never married*), CASIC 4 (*interest in child pornography spanned 2 or more years*), CPORT 5 (*pedophilic or hebephilic interests, clinical diagnosis*), and CASIC 2 (*possessed child pornographic videos*) could prove relevant for future risk assessment tools. The scale constructed here showed better predictive validity than the CASIC and the CPORT with CASIC rating, but would have to prove itself in other samples.

## Data Availability

The datasets presented in this article are not readily available due to the sensitive nature of the research. Requests to access the datasets should be directed to Fritjof von Franqué, f.von-franque@uke.de.

## Footnotes

1 Despite our conviction that a shift in terminology away from ‘child pornography’ is necessary, we employ the term throughout the remainder of this manuscript when referencing specific legal offenses or citing established items from existing risk assessment instruments.

2 Originally, we had used the STATIC-99 (Hanson and Thornton, 1999), but as it overlaps very strongly with the STATIC-C, we decided against using it.

3
https://kein-taeter-werden.de/journalisten/#awb-open-oc__1232
